# Application of synthetic biology in cyanobacteria and algae

**DOI:** 10.3389/fmicb.2012.00344

**Published:** 2012-09-19

**Authors:** Bo Wang, Jiangxin Wang, Weiwen Zhang, Deirdre R. Meldrum

**Affiliations:** ^1^Center for Biosignatures Discovery Automation, The Biodesign Institute, Arizona State UniversityTempe, AZ, USA; ^2^Biological Design Graduate Program, Arizona State UniversityTempe, AZ, USA

**Keywords:** cyanobacteria, algae, synthetic biology, biofuel, green chemistry

## Abstract

Cyanobacteria and algae are becoming increasingly attractive cell factories for producing renewable biofuels and chemicals due to their ability to capture solar energy and CO_2_ and their relatively simple genetic background for genetic manipulation. Increasing research efforts from the synthetic biology approach have been made in recent years to modify cyanobacteria and algae for various biotechnological applications. In this article, we critically review recent progresses in developing genetic tools for characterizing or manipulating cyanobacteria and algae, the applications of genetically modified strains for synthesizing renewable products such as biofuels and chemicals. In addition, the emergent challenges in the development and application of synthetic biology for cyanobacteria and algae are also discussed.

## INTRODUCTION

Cyanobacteria and algae are endowed with the complex photosynthesis systems (Mulkidjanian et al., 2006) which can absorb solar irradiation with a broad wave length and thereafter channel the absorbed energy to other forms of energy carriers such as chemicals (Chisti, 2007; Takahashi et al., 1998; van de Meene et al., 2006) and electricity (Furukawa et al., 2006; Pisciotta et al., 2010). Solar irradiation is a clean, abundant, and renewable energy resource and, if being properly and efficiently transferred, would be more then enough to power the entire human society (Rittmann, 2008). In addition, growing cyanobacteria and algae do not require arable land, which would eventually alleviate the increasing food prices due to the growing crop-based microbial industries (Rittmann, 2008). In contrast, they can fix carbon dioxide (CO_2_), a type of greenhouse gas, during photosynthesis. Furthermore, cyanobacteria and algae grow faster than plants and bear relatively simple genetic background which is relatively easy to manipulate (Koksharova and Wolk, 2002).

As an emerging discipline that tackles biotechnology from a rational-design approach, synthetic biology aims to redesign existing biological systems or create artificial life (Benner, 2003; Endy, 2005; Mukherji and van Oudenaarden, 2009). In recent years, synthetic biology research has been focused on model species such as *Escherichia coli* and yeast, and has greatly boosted not only the in-depth understanding of the biological mechanisms in these cells, but also the capability and efficiency of these systems in biological production of various useful products (Benner, 2003; Lee and Lee, 2003; Martin et al., 2003; Isaacs et al., 2004; Ro et al., 2006; Dwyer et al., 2007; Atsumi et al., 2008b; Inui et al., 2008; Keasling, 2008; Prather and Martin, 2008; Zhang et al., 2008, 2012; Bayer et al., 2009; Ma et al., 2009; Mukherji and van Oudenaarden, 2009; Steen et al., 2010; Yim et al., 2011). However, with over 40 cyanobacterial genome sequences^1^ and more than 60 algal genome sequences^2^ being completed and published, application of synthetic biology in cyanobacteria and algae has significantly lagged behind those in *E. coli* and yeast. Considering the aforementioned inherent merits of the photosynthetic microbes, we believe it would be of great scientific and application values to further develop synthetic biology tools and apply them in cyanobacteria and algae. We herein review the recent progresses and the challenges in developing and applying synthetic biology for cyanobacteria and algae.

## TOOLS FOR SYNTHETIC BIOLOGY IN CYANOBACTERIA AND ALGAE

### DEVELOPMENT OF “BIOBRICKS” FOR CYANOBACTERIA AND ALGAE

“BioBricks” stand for standardized DNA parts with common interface and can be assembled in living organisms. They are the basic interchangeable elements for regulating the genetics^3^. Here we focus on the development of the most common BioBricks for cyanobacteria and algae (i.e., promoters, transtriptional terminators, ribosome binding sites, and other regulatory factors).

#### Promoters

Both native and foreign promoters have been evaluated in cyanobacteria, mostly using *Synechococcus elongatus* PCC 7942 (hereafter *Synechococcus* 7942) and *Synechocystis* sp. PCC 6803 (hereafter *Synechocystis *6803) as model species (**Table 1**). The native promoters used are usually from genes essential to photosynthesis such as CO_2_ fixation (P_rbc__L_, P_cmp_, P_sbt_), photosystem I (PSI; P_psaA,_ P_psaD_), PSII (P_psbA1,_ P_psbA2_), and photosynthesis antenna protein phycocyanin (P_cpc_). A native nickel-inducible promoter, P_nrsB_, has also been successfully utilized to express phage lysis genes in *Synechocystis* 6803 (Liu and Curtiss, 2009). Besides the native promoters, a limited number of foreign promoters have also been characterized in cyanobacteria. The chimeric P_tac_/P_trc_ promoter, a strong promoter in *E. coli*, has been used in *Synechococcus* and *Synechocystis* species to initiate high-level expressions of the interest genes (Geerts et al., 1995; Ng et al., 2000; Atsumi et al., 2009; Huang et al., 2010; Niederholtmeyer et al., 2010; Lan and Liao, 2011). It is noteworthy that the composition of the cyanobacterial holopolymerase is quite different from those in most bacteria (including *E. coli*), so commonly used *E. coli* promoters might perform differently when introduced into cyanobacteria (Heidorn et al., 2011). A recent study on gene expression analysis in *Synechocystis* 6803 showed that the strength of P_trc1O_ (a version of the P_trc_/P_tac_ promoter) was more than fourfold higher than all versions of the promoter of native ribulose bisphosphate carboxylase/oxygenase (RuBisCO) large subunit, P, whereas the common *E. coli* promoters P_lac_, P_tet_, and λ P_R_ exhibited very low or no detectable activities in the same system (Huang et al., 2010). Since currently very little is known about the performance of various native and foreign promoters in cyanobacteria, a systematic investigation on behaviors of various promoters in cyanobacteria would be important.

**TABLE 1:** Selected promoters used in cyanobacteria.

Promoters Sources	Gene(s)	Expression hosts	Reference
P_rbc_	*Synechococcus* 6301, *Synechocystis* 6803, *Synechococcus* 7942	firefly luciferase, *pdc, adh, far, accBCDA, accD, accA, fatB2*	*Synechococcus* 6301, *Synechocystis* 6803, *Synechococcus* 7942	Takeshima et al. (1994), Deng and Coleman (1999), Liu et al. (2011b), Tan et al. (2011)
P_petE_	*Synechocystis* 6803**	*far, far1, far2, accBCDA*	*Synechocystis* 6803**	Tan et al. (2011)
P_psbA1_	*Synechococcus* 7942	*efe, hydEF, hydG, cvrbcLS*	*Synechococcus* 7942	Sakai et al. (1997), Takahama et al. (2003), Ducat et al. (2011a)
P_psbA2_	*Synechocystis* 6803	*pdc, adh, cvrbcLS, ispS, tesA, fatB1, fatB2*	*Synechocystis* 6803, *Synechococcus* 7942**	Iwaki et al. (2006), Dexter and Fu (2009), Lindberg et al. (2010), Liu et al. (2011b)
P_psaA_	*Synechocystis* 6803**	*luxAB*	*Synechocystis* 6803	Muramatsu and Hihara (2006)
P_psaD_	*Synechocystis* 6803**	*luxAB*	*Synechocystis* 6803	Muramatsu and Hihara (2007)
P_cpc_	*Synechocystis* 6714	*luxAB, accB, accC*	*Synechococcus* 7942	Imashimizu et al. (2003)
P_rnpB_	*Synechocystis* 6803**	GFP	*Synechocystis* 6803	Huang et al. (2010)
P_cmp_	*Synechocystis* 6803	*fol, gpl, shl*	*Synechocystis* 6803	Liu et al. (2011a)
P_sbt_	*Synechocystis* 6803	*gpl*	*Synechocystis* 6803	Liu et al. (2011a)
P_nrsB_	*Synechocystis* 6803**	holin, endolysin, auxiliary lysis enzyme	*Synechocystis* 6803	Liu and Curtiss (2009)
P_T7_	Coliphage T7	*luxAB*	*Anabaena* sp. 7120	Wolk et al. (1993)
P_lac_	*E. coli*	*atoB, adhE2, ter, hbd, crt, hydA, alsS, ilvC, ilvD,*	*Synechococcus* 7942	Atsumi et al. (2009), Ducat et al. (2011a), Lan and Liao (2011)
P_trc_/P_tac_	*E. coli*	*petE, atoB, adhE2, ter, hbd, crt, kivd, rbcLS, invA, glf, ldhA, lldP, phrA, *GFP, EYFP**	*Synechococcus* 7942, *Synechocystis* 6803	Geerts et al. (1995), Ng et al. (2000), Atsumi et al. (2009), Huang et al. (2010), Niederholtmeyer et al. (2010), Lan and Liao (2011)
P_tet_	*E. coli*	GFP	*Synechocystis* 6803	Huang et al. (2010)

In algae, CaMV 35S and SV40 promoters from viruses have been used to express target genes (Benfey et al., 1990; Wang et al., 2010). However, the most effective promoters have been derived from highly expressed algal genes. For example, the widely used promoters for *Chlamydomonas* transformation have been derived from the 5′ untranslated region of the *Chlamydomonas reinhardtii* RuBisCO small subunit gene (*rbcS2* ;Stevens et al., 1996), *Chlamydomonas *heat shock protein 70A gene* hsp70A* (Schroda et al., 2000), marine diatom fucoxanthin-chlorophyll a/c binding protein gene *fcp* (Apt et al., 1996; Miyagawa-Yamaguchi et al., 2011), *Dunaliella* duplicated carbonic anhydrase 1 (DCA1; Li et al., 2010; Lu et al., 2011), *Porphyra yezoensis* actin1 gene (PyAct1; Takahashi et al., 2010), and two *Nannochloropsis *unlinked violaxanthin/chlorophyll a-binding protein (VCP) genes, *VCP1* and *VCP2* (Kilian et al., 2011).

#### Transcriptional terminators

Placing a transcription terminator downstream of the introduced genes will prevent effects on the expression of genes adjacent to the insertion loci; meanwhile, placing a terminator upstream of the promoter of an introduced gene will also prevent any background transcription effect on the upstream genes (Adhya and Gottesman, 1982). So far only a few native and foreign terminators have been utilized in cyanobacteria, including the cyanobacterial RuBisCO terminator (Takeshima et al., 1994) and strong *E. coli* terminators such as *rrnB* terminator (Geerts et al., 1995; Takahashi et al., 1998; Atsumi et al., 2009), bacteriophage T7 terminator (Lang and Haselkorn, 1991; Argueta et al., 2004), and *rrnBT1*-*T7TE* double terminator^4^ (Huang et al., 2010). Very little work has been conducted to characterize the termination efficiencies in cyanobacteria and algae.

#### Ribosome binding sites

The ribosome binding sites (RBS) play a crucial role in initiating the translation of downstream target genes. Upon translation initiation, the 3′-terminal sequence of the 16S rRNA interacts with the core Shine–Dalgarno (SD) sequence of RBS by complementary pairing of the nucleic acids. For example, in cyanobacterium *Synechocystis* 6803, the 3′-terminal sequence of the 16S rRNA is AUCACCUCCUUU (Kaneko et al., 1996; Ma et al., 2002) and therefore the optimal complementary SD sequence should be AAAGGAGGUGAU (core SD sequence underlined). Heidorn et al. (2011) studied the efficiencies of different RBS in expressing GFP in *Synechocystis *6803 and found that the RBS sequence UAGUGGAGGU gave about twofold higher translation efficiency than a RBS sequence AUUAAAGAGGAGAAA and about fourfold higher than those of sequences UCACACAGGAAAG and AAAGAGGAGAAA. However, it is found that the efficiency of the same RBS might vary across species, such as *E. coli *vs. *Synechocystis* (Heidorn et al., 2011). The efficiency of a given RBS also depends on the surrounding nucleotide sequence that may result in secondary structures and the spacing between the SD sequence and the translation start codon AUG (de Smit and van Duin, 1990; Chen et al., 1994; Pfleger et al., 2006). In order to predict the translation efficiency of a given RBS in various genetic contexts, Salis et al. (2009) have established a thermodynamic model that calculates the impact from the SD sequence, the start codon, the spacing between the SD sequence and the start codon, and the mRNA secondary structure; the model can accurately predict protein expression levels within a factor of 2.3 over a range of 100,000-fold in *E. coli*. Similar model should be employed to optimize the RBS for gene expression in cyanobacteria.

#### Negative regulation of gene expression

The down-regulation of the target gene expression has been studied at transcriptional, translational, and post-translational levels in cyanobacteria. The negative transcriptional factor, LacI, has been utilized as a repressor in regulating the P_tac_/P_trc_ controlled target gene expression (Geerts et al., 1995; Atsumi et al., 2009; Huang et al., 2010; Niederholtmeyer et al., 2010; Ducat et al., 2011a; Lan and Liao, 2011). However, lacI-P_tac_/P_trc_ expression system can result in severe leaky expression of target genes (Huang et al., 2010). By placing dual *lac* operators upstream of P_tac_/P_trc_ promoter the leaky expression of downstream target genes was significantly repressed; however, this also resulted in a limited induction of the promoter with presence of the inducer IPTG (Huang et al., 2010). Degradation tags which can be fused to the target proteins through genetic engineering have also been investigated in cyanobacteria. Three *ssrA* protease degradation tags including ASV, AAV, and LVA were fused to EYFP and expressed in *Synechocystis* 6803. The results indicated that LVA is the strongest degradation tag, AAV is the weaker one and ASV is the weakest (Huang et al., 2010). Recently, it has been discovered that antisense RNAs (asRNAs) play an important role in cyanobacterial gene regulation (Hernández et al., 2006; Georg et al., 2009; Cerutti et al., 2011; Mitschke et al., 2011). asRNAs thus provide another approach for gene silencing in cyanobacteria. For example, Mussgnug et al. (2007) have successfully down-regulated the expression of light-harvesting antenna complexes through RNA interference.

#### Endogenous enhancers

The transcription of an interest gene can be positively affected by placing in the gene cluster an enhancer, a short DNA fragment which interacts with certain proteins to enhance the transcription. In cyanobacteria, some light-responsive elements exhibit enhancer activities. For example, the 5′-untranslated regions of the *psbAII* and *psbAIII* genes of *Synechococcus* 7942 have been found as enhancers which can increase the expression of downstream genes by 4- to 11-fold when combined with an *E. coli* promoter (*conII*) in the *Synechococcus* 7942 host strain (Li and Golden, 1993). A recent study by Eichler-Stahlberg et al. (2009) showed that inserting three introns from the native alga *C. reinhardtii* *RBCS2* gene into the recombinant luciferase and erythropoietin resulted in up to fourfold increase of the expression levels. By fusing the recombinant luciferase with the endogenous RuBisCO LSU protein, Muto et al. (2009) has achieved enhanced luciferase expression by 33-fold.

### PLASMID VECTORS

Both integrative and replicative plasmids have been developed for cyanobacteria. In cyanobacteria, integrative plasmids are usually utilized as vectors to integrate foreign genes into the cyanobacterial genomes via homologous recombination (Golden et al., 1987; Eaton-Rye, 2004; Heidorn et al., 2011). Integrative plasmids usually cannot replicate themselves and would eventually be eliminated through cell division. Replicative plasmids are those which can replicate in host cyanobacteria and the replication properties can be descended to daughter cells. Replicative cyanobacterial plasmids can be classified into two types: those with replicons of broad-host range plasmids (Mermet-Bouvier et al., 1993; Mermet-Bouvier and Chauvat, 1994; Ng et al., 2000; Huang et al., 2010) and those derived from endogenous cryptic plasmids (Reaston et al., 1982; Wolk et al., 1984; Lang and Haselkorn, 1991; Summers et al., 1995; Deng and Coleman, 1999; Argueta et al., 2004; Iwaki et al., 2006). Representative shuttle vectors for cyanobacteria are listed in **Table 2**. The copy numbers of the broad-host range RSF1010-derived plasmids have been reported as about 10 per chromosome in *E. coli* cells and 10–30 per cell in *Synechocystis* (Ng et al., 2000; Huang et al., 2010) which is slightly higher than the average copy number (approximately 10) of the *Synechocystis* chromosome (Eaton-Rye, 2004). Due to lack of an active partitioning mechanism, RSF1010-derived plasmids tend to be eliminated in cells and thus antibiotic selection pressure is required for the maintenance (Becker and Meyer, 1997; Meyer, 2009).

**TABLE 2:** Representative shuttle vectors for cyanobacteria.

Cyanobacterial replicons	*E. coli* replicons	Representative vectors	Host cyanobacteria	Reference
pDU1	pMB1	pRL1, pJL3	*Anabaena* 7120, *Anabaena* 7118, *Anabaena* M-131, *Nostoc *7524	Reaston et al. (1982), Wolk et al. (1984), Lang and Haselkorn (1991)
pDC1	pMB1	pSCR119/202, pSUN119/202	*Nostoc* sp. MAC 8009, *Nostoc punctiforme*, *Nostoc* ATCC 29133	Lambert and Carr (1983), Summers et al. (1995), Argueta et al. (2004)
pUH24	P15A	pUC303	*Synechococcus* 7942	Kuhlemeier et al. (1983), Iwaki et al. (2006)
pUH24	pMB1	pCB4, pSG111	*Synechococcus* 7942	Golden and Sherman (1983), Luinenburg and Coleman (1993), Deng and Coleman (1999)
pAQ1	pMB1	pAQE17	*Synechococcus* 7002	Buzby et al. (1985)
PBA1	pMB1	pARUB19	*Synechococcus* 6301	Takeshima et al. (1994)
RSF1010	RSF1010	pFC1, pSL1211, pPMQAK1,	*Synechosystis* 6803, *Synechosystis* 6714, *Synechococcus* 7942, *Synechococcus* 6301, *Anabaena* 7120, *Nostoc* ATCC 29133	Mermet-Bouvier et al. (1993), Mermet-Bouvier and Chauvat (1994), Ng et al. (2000), Huang et al. (2010)

Plasmid vectors have been developed to transform algae (León-Bañares et al., 2004). Recombinant eukaryotic algal viruses as transformation vectors (Langridge et al., 1986) and *Agrobacterium tumefaciens*-mediated method (Kumar et al., 2004) were also successfully developed for both marine and freshwater algae (Wang et al., 2010).

### CODON USAGE

Since different organisms usually bear particular codon usage patterns, when a gene is cloned from one species and expressed in a second organism, some codons might become rare codons in the new host, leading to poor translation efficiency (Kane, 1995). Genes in cyanobacteria show a bias in use of synonymous codons (Campbell and Gowri, 1990; Nakamura et al., 2000; Beck et al., 2012; Yu et al., 2012); it is thus very important to examine the difference in codon usage of a heterologous gene before it is expressed in cyanobacteria. In a recent study, Lindberg et al. (2010) studied the effects of codon usage on heterologous expression of kudzu *IspS* gene (encoding the isoprene synthase) in *Synechocystis* 6803. The results showed that the codon-optimized *IspS* showed remarkable improved expression, 10-fold higher than that of the native *IspS* gene under control of the same promoter. The importance of codon optimization in algal genetic applications is also increasingly acknowledged. For instance, it has been shown that codon bias significantly affects the GFP expression in *C. reinhardtii* (Heitzer et al., 2007). As a result, in recent transgenic research, the codon-optimized luciferase gene was used in a green alga *Gonium pectorale *(Lerche and Hallmann, 2009) and the codon-modified β-glucuronidase gene was transformed in a red seaweed *P. yezoensis *(Takahashi et al., 2010).

### TRANSFORMATION OF CYANOBACTERIA AND ALGAE

Methods to introduce DNA into cyanobacteria include conjugation (Thiel and Wolk, 1987; Elhai and Wolk, 1988), electroporation (Zang et al., 2007), and natural transformation (Shestakov and Khyen, 1970; Grigorieva and Shestakov, 1982; Kuhlemeier and van Arkel, 1987). The methods have been well summarized in several recent reviews (Eaton-Rye, 2004; Koksharova and Wolk, 2002; Heidorn et al., 2011) and we suggest readers to refer these excellent reviews for details. Compared to cyanobacteria, transformation methods for algae are less developed and more complicated. Since the chloroplast and nucleus of alga *C. reinhardtii* were stably transformed more than two decades ago (Boynton et al., 1988; Debuchy et al., 1989; Fernández et al., 1989), different methods have been employed in algal transformation which include, but not limited to, particle bombardment, glass bead agitation, microinjection, electroporation and *A. tumefaciens*-mediated transformation (León-Bañares et al., 2004; Coll, 2006; León and Fernández, 2007; Potvin and Zhang, 2010). Specifically, bombardment of target cells with DNA-coated metal particles turns to be an effective and highly reproducible method to transform algae. This method has been so far applied in the transformation of nuclear and chloroplast of many algal species such as *C. reinhardtii*, *Volvox carteri*, *Chlorella sorokiniana*, *Chlorella ellipsoidea*, *Chlorella kessleri*, *Haematococcus pluvialis,* *Phaeodactylum tricornutum*, and *G. pectorale* (Boynton and Gillham, 1993; Potvin and Zhang, 2010). In addition, agitation of the cell wall-deficient algal cells with glass beads, polyethylene glycol (PEG) and foreign DNA has been used to transform algae such as *C. reinhardtii*, *Dunaliella salina*, and red alga *Porphyra haitanensis* (Kindle, 1990; Feng et al., 2009; Wang et al., 2010). Microinjection of the viral SV40 DNA or the chimeric construction pSV2neo into the marine unicellular green alga *Acetabularia mediterranea* also resulted in a high yield and stable nuclear transformation (Neuhaus et al., 1986); nevertheless, it is hard to operate and the throughput of transformation is low. *Agrobacterium tumefaciens* has been used to mediate the transformation of *C. reinhardtii* (Kumar et al., 2004) and *H. pluvialis* (Kathiresan et al., 2009). Recently, it was discovered that the industrially relevant oil-producing alga *Nannochloropsis* sp. is haploid and can be transformed with high efficiency using high electric field electroporation. It has also been found that efficient stable transformation of this species via homologous recombination requires using linear DNA fragment rather than circular plasmid DNA (Kilian et al., 2011). However, the mechanism for the high homologous recombination efficiency is to be elucidated.

## APPLICATIONS OF MODIFIED CYANOBACTERIA AND ALGAE

We focus here on recent progress in producing biofuels and other useful chemicals using genetically modified cyanobacteria and algae. For other applications, readers can refer to several other excellent reviews published recently (Radakovits et al., 2010; Ruffing, 2011; Qin et al., 2012).

### BIOFUELS

**FIGURE 1:**
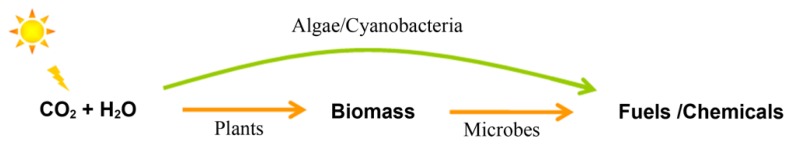
**Routes for biological production of fuels and chemicals.** Arrows indicate the carbon and energy flow between different carriers.

United States consumed 13.3 million barrels of petroleum per day for transportation purposes in 2009, accounting for 71% of all petroleum used (Energy Information Administration, 2010). Many alternatives to current liquid fuels have been proposed, including ethanol, 1-butanol, isobutanol, short-chain alcohols, short-chain alkanes, biodiesel (FAME, fatty acid methyl esters), fatty alcohols, alkanes, linear and cyclic isoprenoids (Lee et al., 2008; Connor and Atsumi, 2010). Current routes for biological production of fuels and chemicals are summarized in **Figure 1**. Traditionally people follow a two-step route to firstly collect plant biomass and then convert biomass to fuels by microbial fermentation (Stephanopoulos, 2007); whereas recently interest in harnessing photosynthetic microbes to directly convert CO_2_ to fuels has been dramatically increased (Chisti, 2007; Lu, 2010). Compared to crops, the per-hectare oil yield of cyanobacteria or microalgae is about two orders of magnitude higher and the cultivation land needed is around two orders of magnitude less (Chisti, 2007). It is estimated that more than $1 billion has been invested in the algae-to-biofuel research and development since 2007 in US alone (Mascarelli, 2009).

#### Biodiesel

Cyanobacteria and algae are rich in energy stock compounds, such as diacylglycerol (DAG), triacylglycerol (TAG) and starch, which can be extracted and used for biodiesel production (van de Meene et al., 2006; Chisti, 2007; Radakovits et al., 2010; Sheng et al., 2011). To further increase the oil contents in the cells, effects have been made to block metabolic pathways as well as to overexpress genes of limiting steps. For example, two different starch-deficient strains of *C. reinhardtii*, the *sta6* and *sta7* mutants that carries gene knockout in the ADP-glucose pyrophosphorylase and isoamylase genes, respectively, have been isolated (Mouille et al., 1996; Posewitz et al., 2005); and these mutants accumulated increased levels of TAG during nitrogen deprivation (Wang et al., 2009). Another starchless mutant of *Chlorella pyrenoidosa* has also been reported that the lipid content of this mutant has been elevated by nearly twofold relative to the wild-type under nitrogen limitation culture conditions (Ramazanov and Ramazanov, 2006). It indicated that blocking the starch biosynthesis may be an effective way to increase lipid, and thus potentially biodiesel, production.

Nevertheless, lipid extraction process is energy-intensive and significant amount of glycerol as a byproduct have been two of the major hurdles for commercial production of biodiesel (Chisti, 2007; Fernando et al., 2007; Liu and Curtiss, 2009). Efforts have been made from both process engineering and genetic engineering approaches to facilitate the lipid extraction (Liu and Curtiss, 2009; Liu et al., 2011a; Sheng et al., 2011). Specifically, Liu and colleagues have constructed inducible systems to conditionally express phage lysis genes and lipolytic enzyme genes in *Synechocystis* 6803 to trigger the cell lysis upon harvest and thus help lipid extraction from this species (Liu and Curtiss, 2009; Liu et al., 2011a). To produce secretable biofuels from a synthetic biology approach is another way to resolve above issues.

#### Free fatty acids

Enhanced production of free fatty acid (FFA) has already been achieved in *E. coli* through a series of genetic engineering (Davis et al., 2000; Lu et al., 2008). In a recent study, Liu et al. (2011b) engineered cyanobacterium *Synechocystis* strains to produce and secret FFAs to up to 197 mg/L at a cell density of 1.0 × 10^9^ cells/mL. The acetyl-CoA carboxylase (ACC) was overexpressed to drive the metabolic flux toward FFAs, while the fatty acid activation gene *aas* (*slr1609*) was deleted to inactivate the FFAs degradation. Poly-β-hydroxybutyrate (PHB) synthesis genes (*slr1993* and *slr1994*) and the phosphotransacetylase gene *pta* (*slr2132*) were deleted to block competitive pathways. Particularly, two genetic modifications turned to significantly increase the FFAs production and secretion: overexpression of thioesterases and weakening the polar peptidoglycan layer of the cell wall of *Synechocystis* 6803.

#### Alkanes and alkenes

Although it was known that some cyanobacteria can synthesize alkanes, the molecular mechanism had been mysterious until recently an alkane/alkene biosynthetic pathways were identified in cyanobacteria (Steen et al., 2010; Mendez-Perez et al., 2011). Steen et al. (2010) identified an alkane/alkene biosynthetic pathway that two successive biochemical reactions catalyzed by an acyl-ACP reductase and an aldehyde decarbonylase, respectively, converts acyl-ACP (intermediates of fatty acid metabolism) to alkanes/alkenes. In order to increase the alkane production in cyanobacteria, heterologous expression of acyl-ACP reductase and aldehyde decarbonylase genes (from *Synechococcus* 7942) has been achieved in *Synechococcus* 7002, which led to a total intracellular accumulation of n-alkane to up to 5% of the dry cell weight (Reppas and Ridley, 2010). In another research, Mendez-Perez et al. (2011) identified the genes responsible for α-olefin biosynthesis in *Synechococcus* 7002. In addition, overexpression of the *accBCDA* operon (which encodes ACC) in *Synechocystis* was also reported to enhance alkane/alkene production (Tan et al., 2011), consistent with the aforementioned results of FFAs production. Although it is believed there are certain alkane/alkene secretion pathways, the specific mechanisms are still under exploration (Radakovits et al., 2010).

#### Ethanol

Ethanol production via microbial fermentation has undergone a sharp increasing in the past decade for its utility as supplement in transportation fuel (Stephanopoulos, 2007; Energy Information Administration, 2010). In 1999, photosynthetic production of up to 230 mg/L ethanol has been reported using genetically engineered cyanobacterium *Synechococcus* 7942, in which an artificial operon of *pdc-adh* (genes originally from *Zymomonas mobilis*) was expressed under a P_lac_ and P_rbc_ promoters via a shuttle vector pCB4 (Deng and Coleman, 1999). In a recent study, the *pdc-adh* expression cassette was integrated into the chromosome of *Synechocystis* 6803 at the *psbA2* locus. Driven by the light-inducible strong P_psbA2_ promoter, expression of *pdc/adh* resulted in ~550 mg/L ethanol production by the engineered *Synechocystis* under high light (~1000 μE/m^2^/s) conditions (Dexter and Fu, 2009). In algae, although many species have fermentative pathways to produce ethanol, the pathways are only functional under dark and anaerobic conditions (Hirayama et al., 1998). Algal ethanol is currently produced via heterotrophic fermentation of algal biomass using heterotrophs such as yeast and *E. coli* (Nguyen et al., 2009; Harun et al., 2010; Wargacki et al., 2012), which follows the two-step route (**Figure 1**). Direct photosynthetic production of ethanol by algae would be possible using a similar approach as being demonstrated in cyanobacteria by expressing foreign ethanol biosynthesis pathways, or by tuning the native regulatory pathways in algae.

#### Isobutanol and 1-butanol

Compared with ethanol, isobutanol and 1-butanol have much higher energy density. The energy density of butanol reaches 29.2 MJ/L, about 90% of that of gasoline, 32.5 MJ/L, and it is also less volatile and less corrosive than ethanol (Dürre, 2007). Therefore, butanol is regarded as a better gasoline substitute. Recently, significant progress has been achieved for photosynthetic production of butanol. Liao and colleagues introduced an artificial isobutanol biosynthesis pathway into *Synechococcus* 7942 and the engineered strains were able to photosynthetically produce isobutyraldehyde and isobutanol at titers of 1100 and 450 mg/L, respectively (Atsumi et al., 2009). In contrast, photosynthetic production of 1-butanol in oxygenic cyanobacteria or algae has been hard because the intrinsic oxygen-sensitivity and NADH-dependence of the 1-butanol biosynthetic pathway are conflict with the photo-oxygenisis and lack of NADH cofactors in cyanobacteria (Atsumi et al., 2008a; Inui et al., 2008; Lan and Liao, 2011). When a 1-butanol pathway was overexpressed in *Synechococcus* 7942, the 1-butanol was barely detectable (~1 mg/L) after 2 weeks cultivation under photosynthetic conditions. Up to 14.5 mg/L 1-butanol has been achieved in *Synechococcus* 7942 under an anoxic condition (Lan and Liao, 2011). Further analysis revealed that the reversible acetyl-CoA condensation reaction catalyzed by thiolase (encoded by *atoB*) strongly favors the thiolysis of acetoacetyl-CoA rather than the condensation of two acetyl-CoA molecules, and thus AtoB may be insufficient to drive the flux from acetyl-CoA pool toward 1-butanol biosynthesis under photosynthetic conditions (Lan and Liao, 2012). To this end, an alternate ATP-driven acetoacetyl-CoA biosynthetic pathway was constructed by overexpressing an acetoacetyl-CoA synthase (NphT7) which instead condenses malonyl-CoA and acetyl-CoA in *Synechococcus*. With co-expressing the downstream NADH-dependent 1-butanol biosynthetic pathway, 6.5 mg/L 1-butanol has been produced under photosynthetic conditions. After the NADH-dependent bifunctional aldehyde/alcohol dehydrogenase (AdhE2) was further replaced with separate NADPH-dependent butyraldehyde dehydrogenase (Bldh) and alcohol dehydrogenase (YqhD), the 1-butanol production was increased by fourfold, up to ~30 mg/L, under the same photosynthetic condition (Lan and Liao, 2012).

#### Longer carbon chain fatty alcohols

In order to produce long-chain alcohols, Lu and colleagues heterologously expressed fatty acetyl-CoA reductases from different sources in *Synechocystis* and the resultant strains achieved production of fatty alcohols, including hexadecanol (C16) and octadecanol (C18; Tan et al., 2011). Although the titer was very low (about 0.2 mg/L), it is amenable for further improvement via further enhancing upstream pathways and addressing secretion issues as that in the engineering of *Synechocystis* 6803 for enhanced fatty acid production (Liu et al., 2011b). Production of the intermediate-chain alcohols (C5 to C10) in *E. coli* has been well summarized by Lamsen and Atsumi (2012). Briefly, C5 to C10 alcohols have been successfully biosynthesized via the expanded 1-butanol pathway (Dekishima et al., 2011), the engineered reversal of the β-oxidation pathway (Dellomonaco et al., 2011) and the 2-keto acid metabolic pathways (Atsumi et al., 2008b; Zhang et al., 2008). Since cyanobacteria and algae share with *E. coli* the most chassis metabolic pathways required for longer-chain alcohol biosynthesis, it is believed that similar approaches can be used to achieve the biosynthesis of alcohols with carbon chain length >5 in cyanobacteria and algae.

#### Hydrogen

Besides liquid biofuels, production of hydrogen – a gaseous, carbon-free, and high-energy-content fuel – in algae and cyanobacteria has also gained increasing attention in recent years (Melis et al., 2000; Kruse et al., 2005; Ghirardi et al., 2007, 2009; Hankamer et al., 2007; Hemschemeier et al., 2009; Lee et al., 2010; Srirangan et al., 2011). Many cyanobacteria and algae naturally produce hydrogen as a secondary metabolite to balance the redox energetics. In order to fortify the hydrogen production, endeavor has been made to augment the electron flux, instead of the carbon flux, toward H_2_ biosynthesis catalyzed by hydrogenases (2H^+^ + 2e^–^ → H_2_). In alga *C. reinhardtii*, for instance, blocking the cyclic electron transfer around PSI turned to eliminate the possible electron competition for electron with hydrogenase; as a result, the H_2_ evolution rate increased 5–13 times under a range of conditions (Kruse et al., 2005). Hydrogenase has been tethered to the PSI to obtain a much greater electron throughput and thus H_2_ evolution rate (Ihara et al., 2006; Schwarze et al., 2010; Lubner et al., 2011). However, to date these experiments were all conducted *in vitro* and efforts need to be made from a synthetic biology approach to validate the concept *in vivo*. In another study, expression of an exogenous ferredoxin from *Clostridium acetobutylicum* in addition to the native ferredoxin could fortify the electron flow toward the hydrogenase HydA via siphoning electrons from the fermentation of internal reducing equivalents (such as glycogen). As a result, the hydrogen production was enhanced by approximately twofold (Ducat et al., 2011a) under light-dependent anoxic conditions. On the other hand, efforts have been made to block pathways competitive for reductant consumption to facilitate the H_2_ production. For example, after the *ldhA* gene (which is responsible for NADH consumption in lactate production) was inactivated in* Synechococcus* 7002, the NADH/NAD^+^ ratio increased markedly and therefore the hydrogen production by the native bidirectional [NiFe] hydrogenase was increased fivefold under anoxic dark conditions (McNeely et al., 2010). The oxygen-sensitivity of both two major [NiFe] and [FeFe] hydrogenases is the greatest challenge to date which is discussed infra.

### OTHER COMMODITY CHEMICALS

Although significant attention has been paid to photosynthetic production of fuels from CO_2_, the relative values (in term of USD per photon) of fuels are much lower than those of other commodity chemicals. For example, it is estimated that the relative value of a photon fixed in lactic acid is about 3.5-fold greater than that in octane (Ducat et al., 2011b). Therefore, photosynthetic production of chemicals with higher unit values than fuels is economically more desirable at least in the near term.

#### Ethylene

Ethylene, the simplest unsaturated alkene, is one of the most important building-blocks in synthetic chemical industry. However, its production almost exclusively relies on petroleum. To make the production sustainable, biosynthesis of ethylene from renewable resources has been explored. Sakai et al. (1997) first demonstrated photosynthetic production of gaseous ethylene from CO_2_ in genetically engineered *Synechococcus* by heterologously expressing a single *efe* gene of *Pseudomonas syringae* on a pUC303-derived shuttle vector. Later, by integrating the* efe* gene into the *psbA1* locus of the *Synechococcus* 7942 genome, the research group achieved higher ethylene production with a titer of ~37 mg/L (Takahama et al., 2003). However, the engineered *Synechococcus* strains were not genetically stable, resulting in declined ethylene production during successive batch cultivations (Takahama et al., 2003). Since production of every two molecules of ethylenes consumes three molecules of 2-oxoglutarate and one molecule of L-arginine (Fukuda et al., 1992), the genetic instability might be attributed to the shortage in the tricarboxylic acid (TCA) cycle intermediates, leading to a severe depression on cell growth (Takahama et al., 2003). In order to sustain the ethylene production in cyanobacteria or algae, metabolic flux toward TCA cycle should be enhanced and alternative ethylene biosynthesis pathways might be considered (Yang and Hoffman, 1984; Fukuda et al., 1989; Kende, 1993; Kosugi et al., 2000).

#### Isoprene

Isoprene is another important feedstock in the synthetic chemistry and potentially a biofuel. Biosynthesis and emission of isoprene occurs in many plants as a way to cope with heat flecks and reactive oxygen species, and the genetic mechanism has been investigated (Sharkey et al., 2008). Lindberg et al. (2010) cloned the *IspS* gene (encoding**isoprene synthase) from *Pueraria montana* and integrated into the *psbA2* locus of the *Synechocystis* genome, conferring heterologous expression of the isoprene synthase under the light-dependent P_psbA2_ promoter in *Synechocystis*. Codon usage turned to be a very important factor for optimal expression of the *IspS* gene. After codon optimization, the *IspS* gene expression was enhanced by about 10-fold. Isoprene was eventually produced at a rate of ~50 mg/g dry cell/day under high light (~500 μE/m^2^/s) culture conditions. It is noteworthy that heterologous expression of *IspS* by replacing the *psbA2* gene did not affect photosynthesis significantly and depress the growth of the transformants (Lindberg et al., 2010), which was differed from the aforementioned ethylene-producing cyanobacteria (Sakai et al., 1997; Takahama et al., 2003).

#### Acetone

Acetone represents the simplest ketone which serves as a solvent and precursor for industrial chemicals (Yurieva et al., 1996). Microbial production of acetone has been achieved in fermentation of *Clostridia* and recombinant *E. coli *using sugar as feedstocks (Bermejo et al., 1998). However, the maximal yield is merely 50% with the other half carbon being released as CO_2_ when hexose is the sole carbon source. Recently, through a combination of co-expression of the acetoacetate decarboxylase (*adc*) and coenzyme A transferase (*ctfAB*) and deletion of the PHB polymerase (PhaEC) in *Synechocystis *6803, 3–5 mg/L acetone has been produced under nitrogen and phosphate deprived, dark and anaerobic culture conditions. After deleting the phosphotransacetylase-encoding gene *pta*, the competitive acetate production was remarkably reduced and the acetone titer has been evidently increased to 36.0 mg/L in the culture (Zhou et al., 2012).

#### Poly-β-hydroxybutyrates

Cyanobacteria are the natural producers of PHB, a type of polyhydroxyalkanoates (PHAs) that serves as biodegradable plastics (Hein et al., 1998; Taroncher-Oldenburg et al., 2000). However, the yield is very low and nutrient deprivation and acetate addition are usually necessary for accumulation of PHB (Wu et al., 2001). By introducing PHB biosynthesis genes from *Ralstonia eutropha* into *Synechococcus* 7942 coupled with nitrogen starvation and acetate supplementation, the PHB biosynthesis in the recombinant cyanobacteria has reached a maximum of 25.6% of the dry cell weight (Takahashi et al., 1998). Efforts in identifying gene disruptions which might contribute to increase of PHB accumulation were also made and several gene disruptions with positive effects were discovered (Tyo et al., 2009). Nevertheless, similar with other types of macromolecules PHB can not be secreted out of cells; the required extraction process is energy-intensive and remains as one of the major hurdles for commercial applications (Chisti, 2007; Liu and Curtiss, 2009). As a result, 3-hydroxybutyrate (3HB), the monomer of PHB and a building block molecule for other PHAs, has been successfully produced and secreted by genetically engineered *E. coli* (Lee and Lee, 2003; Liu et al., 2007; Tseng et al., 2009). Hence, photosynthetic production of 3HB in cyanobacteria and algae might be a feasible approach to cope with the secretion problem.

#### Lactic acid

Lactic acid is another chemical that can serve as a building block for synthesizing biodegradable polyesters with valuable medical properties. It is also used as a preservative and acidulant in food industry, and can serve as an advanced nutrient for neuron cells (Wee et al., 2006). While conventional production of lactic acid relies on microbial fermentation of sugars (Wee et al., 2006), photosynthetic production of lactic acid using CO_2_ as carbon source has been recently demonstrated (Niederholtmeyer et al., 2010). Through heterologously expressing three genes, including *ldhA*, *lldP*, and *udhA*, in cyanobacterium *Synechococcus* 7942, Niederholtmeyer et al. (2010) has accomplished production of lactic acid with a titer of ~56 mg/L under photoautotrophic culture condition. While LdhA catabolizes the conversion of pyruvate to lactate, expression of the lactate transporter gene *lldP* turned to be essential for lactate secretion from the engineered *Synechococcus* strain (Niederholtmeyer et al., 2010). Repletion of NADH, a cofactor for LdhA, through expressing the NADPH/NADH transhydrogenase (encoded by *udhA*) greatly enhanced the lactate production but reduced the growth rate of *Synechococcus* (Niederholtmeyer et al., 2010).

#### Sugars

Fresh water cyanobacteria accumulate solutes such as glucosylglycerol and sucrose when they are exposed to salt stress (Hagemann, 2011). By knocking out the *agp* gene (which contributes to the biosynthesis of glucosylglycerol) from the *Synechocystis* 6803 genome, Miao et al. (2003) achieved sucrose accumulation of up to 44 mg/L/OD_730_ after 0.9 M salt shock for 96 h. In another study, overexpression of *invA*, *glf*, and *galU* genes in *Synechococcus* 7942 resulted in up to 45 mg/L total hexose production (including glucose and fructose) in the culture supplemented with 200 mM NaCl (Niederholtmeyer et al., 2010). While InvA catalyzes the conversion of sucrose to glucose and fructose, expression of the glucose or fructose transporter GLF (encoded by *glf* gene) was essential for glucose or fructose secretion. Additional expression of GalU enhanced the biosynthesis of intracellular precursors and thus further increased the hexose sugar production by over 30% in the culture (Niederholtmeyer et al., 2010).

## CHALLENGES AND OPPORTUNITIES OF SYNTHETIC BIOLOGY IN CYANOBACTERIA AND ALGAE

Despite of promising progresses, there are challenges ahead for synthetic biology to reach its full power in modifying cyanobacteria and algae for biotechnological applications. Here we briefly discuss the challenges and possible strategies.

### IMPROVING TOOLS FOR GENETIC MANIPULATION

#### Effective “BioBricks”

Although a few “BioBricks” have been characterized in cyanobacteria, the limit number of gene expression elements would not fulfill the need of synthetic biology in cyanobacteria. After an initial gene expression, a fine-tuning of the gene expression is usually the next step in order to further optimize the properties of the genetically engineered strains, which requires a good number of “BioBricks.” Currently most of the “BioBricks” were collected from *E. coli*, but the *E. coli* “BioBricks” might behave differently in cyanobacteria. For example, tightly regulated IPTG-inducible lacI/P_tac_ gene expression system does not work as well in cyanobacteria as it does in *E. coli* (Huang et al., 2010). Thus, systematic collection and characterization of “BioBricks” in cyanobacteria is necessary. Additionally, in contrast to various commercialized *E. coli* and yeast strains that have been genetically modified to serve as chassis for different purposes, there are few such cyanobacterial or algal species available nowadays. To design and construct a series of chassis strains is thus an urgent task. Moreover, to our knowledge, there has been no study of the performance of a given BioBrick in different cyanobacterial species. We assume that a defined BioBrick might behave differently across cyanobacterial species and the efficiency of the BioBrick need to be characterized for each cyanobacterial species.

#### Improved transformation efficiency

Standardized transformation vectors/protocols have been established for model cyanobacteria, such as *Synechococcus* and *Synechocystis*, although the transformation efficiency still needs further improvement (Eaton-Rye, 2004; Heidorn et al., 2011). However, the transformation methods for model filamentous cyanobacteria, such as *Anabaena* and *Spirulina*, are still under development (Ducat et al., 2011b), and so far no genetic engineering has been conducted in the marine N_2_-fixing cyanobacterium *Trichodesmium* despite significant interest on its ability of peaking the fixation of CO_2_ and N_2_ simultaneously during the day time (Chen et al., 1998; Berman-Frank et al., 2001). *In vivo* restriction activities have been demonstrated as an important barrier for introducing foreign DNA into cyanobacterial cells (Elhai et al., 1997; Koksharova and Wolk, 2002). Hence, it would be helpful to construct methylation-defect cyanobacterium host strains or to establish *in vitro *systems that can methylate the foreign DNA before transformation. Additionally, since the bacteriophage λ recombination system has greatly improved the *E. coli* transformation efficiency (Yu et al., 2000), we propose that high-efficiency homologous recombination in cyanobacterial cells might be achievable by developing a proper cyanophage recombination system.

In order to improve transformation efficiency of other algal species, endeavor could be made to uncover the mechanism behind the recently discovered truth that highly efficient homologous recombination occurs after electroporation of the industrially relevant oil-producing alga *Nannochloropsis* sp. (Kilian et al., 2011). Recently, through an approach of *ex vivo *assembly of the chloroplast genome before bombarding it into the green alga *C. reinhardtii*, O’Neill et al. (2012) demonstrated that simultaneous and multi-loci genetic modifications of the chloroplast of the green alga *C. reinhardtii *could occur after one single round of transformation, providing an alternative method to improve the efficiency of multiple-gene transfer.

### IMPROVING PHOTOSYNTHESIS EFFICIENCY

Although the solar energy conversion efficiencies of algae and cyanobacteria are 2–3 folds higher than those of crop plants, the efficiencies are still low with yields around 5–7% during the growing season and around 3% in bioreactors on an annual basis (Blankenship et al., 2011). A recent study on *in silico* modeling of the reconstructed photosynthetic process revealed that the regulation of the photosynthesis activity is quite complex and a high degree of cooperativity of nine alternative electron flow pathways is responsible for optimized photosynthesis performance in *Synechocystis* 6803 (Nogales et al., 2012).

#### Light harvesting

The photosynthetic microorganisms in nature have been selected by the abilities to reproduce but not by the ability to produce a maximal amount of biomass or specific products. In order to thrive in the wild environment, cyanobacteria and algae have maximized their expression of pigments and antenna to compete with the competitors for sunlight. However, when monoculture was employed to produce high-density of biomass or maximal titers of specific products in photo-bioreactors, excessive photon capture by the cells in the surface layer can block the light availability to the cells underneath (Melis, 2009). To address this issue, studies have been conducted on minimizing the size of the photosystem antenna complex through various strategies, such as by expressing truncated light-harvesting antenna complex (LHC) mutants (Blankenship et al., 2011; Ort and Melis, 2011; Work et al., 2012), by down-regulating the expression of LHC through RNA interference (RNAi) and expression of LHC translation repressor in both cyanobacteria and algae (Mussgnug et al., 2007; Work et al., 2012). For example, the photosynthetic activity (measured by oxygen evolution) was about threefold higher in the alga strain* Stm3LR3* (with LHC being down-regulated via RNAi) than in the parent strain *Stm3* (without RNAi) after 100 min of high-light treatment; the cell growth rate also increased under high-light conditions after the LHC was down-regulated via RNAi (Mussgnug et al., 2007). Another bold proposal was to increase the photosynthesis efficiency by extending the light absorption range of the photosystems in cyanobacteria and algae (Blankenship et al., 2011). As the chlorophyll, carotenoids, and other accessory pigments in cyanobacteria and algae capture only visible region of the spectrum of solar radiation (400 to 700 nm), about 50% of the incident solar energy is dissipated and wasted during photosynthesis. Moreover, since the two photosystems compete for light with the same wavelengths, the overall efficiency is significantly reduced. Thus, it was proposed that one of the two photosystems be engineered to extend the absorption maxima to ~1100 nm, approximately doubling the solar photon capture, by heterologously expressing bacteriochlorophyll *b *(Blankenship et al., 2011).

#### CO_2_ fixation

RuBisCO is an essential enzyme in photosynthetic carbon fixation in Calvin–Benson–Bassham (CBB) cycle, catalyzing the combination of ribulose-1,5-bisphosphate with CO_2_. However, the reaction is slow. In addition, RuBisCO can also take O_2_ as substrate in addition to CO_2_ which further lower the carbon fixation efficiency. A recent study has revealed that despite slow catalytic turnover and confused CO_2_/O_2_ substrate specificity, RuBisCOs might have been nearly perfectly optimized (Tcherkez et al., 2006). In nature, cyanobacteria and some algae have evolved certain CO_2_-concentrating mechanisms (CCMs) to increase the CO_2_ fixation efficiencies. In cyanobacteria, RuBisCOs are sequestered together with carbonic anhydrous in carboxysomes, polyhedral microcompartments (MCPs) with proteinaceous shells. Anhydrase catalyzes the conversion of HCO_3_^-^ to CO_2_ which is trapped by MCPs for RuBisCOs. Because CCMs can result in much higher CO_2_ concentration, and thus higher CO_2_ to O_2_ ratio, around the RuBisCOs, the carbon fixation efficiency is greatly increased (Espie and Kimber, 2011). It has been found that *Synechococcus* 7942 cells with more carboxysomes exhibited higher CO_2_ fixation rates (Savage et al., 2010). Heterologous expression of* Synechococcus* 6301 *rbcLS* (that encodes RuBisCO) in *Synechococcus* 7942 also led to more efficient CO_2_ fixation and higher yield of isobutyraldehyde in the genetically modified isobutyraldehyde-producing strain (Atsumi et al., 2009). Besides, overexpression of bicarbonate transporters has also been proposed to improve the photosynthesis efficiency (Price et al., 2011). Alternatively, RuBisCO-independent carbon fixation pathways have been posited. A recent work using *in silico* modeling of the recombination of existing metabolic building blocks showed that some of the proposed carbon fixation cycles have overall higher kinetic rates (Bar-Even et al., 2010). For example, by coupling the phosphoenolpyruvate carboxylase and the core of the natural C4 carbon fixation cycle, the overall CO_2_ fixation rate was predicted as 2–3 folds higher than that of the CBB cycle which employs RuBisCO (Bar-Even et al., 2010).

### OVERCOMING THE OXIDATIVE STRESS

Since cyanobacteria and algae are oxygenic microorganisms, the abundant oxygen evolved by splitting water during photosynthesis process becomes an issue for expressing oxygen-sensitive enzymes. For example, either [NiFe] or [FeFe] hydrogenase required for biological production of H_2_ has low oxygen-tolerance (Lee et al., 2010); and the nitrogenases which fix N_2_ into NH_4_^+^ are also extremely oxygen-sensitive (Fay, 1992). From a broader prospect, this oxygen sensitivity issue could be crucial for successful expression of a large number of pathways from anaerobic microorganisms in oxygenic cyanobacteria and algae. To address the issue, efforts have been made to obtain oxygen-resistant enzymes from nature or through mutagenesis. For example, hydrogenases with better oxygen-tolerance have been found from *Ralstonia eutropha* H16 (Saggu et al., 2009) and *Hydrogenovibrio marinus* (Yoon et al., 2011); and elevated oxygen-tolerance has been made for the hydrogenase of *Desulfovibrio fructosovorans* by a single V74M mutation (Dementin et al., 2009). Alternatively, temporal segregation of oxygenic photosynthesis and hydrogen biosynthesis would be another option. In nature, many cyanobacterial species have evolved the mechanism to photosynthetically fix CO_2_ during day time while fix N_2_ by the oxygen-sensitive nitrogenases during night (Fay, 1992). Thereby, the solar energy can be firstly fixed into carbohydrates, such as starch, during oxygenic photosynthesis and then be utilized to power the oxygen-sensitive reactions during dark anoxic conditions. In addition, spatial segregation could be used. Hydrogenases can be localized to certain advantageous space, such as being expressed in heterocysts, to avoid the oxidative stress (Fay, 1992). Recent studies on bacterial MCPs assembling might have provided another opportunity to spatially segregate incompatible oxygenic and oxygen-sensitive processes (Fan et al., 2010; Heinhorst and Cannon, 2010; Bonacci et al., 2012). Moreover, Mehler reaction can be used to overcome the oxidative stress (Mehler, 1951; Asada, 2006). Mehler reaction has been evolved to overcome the intracellular oxidative stress by scavenging reactive oxygen species in cyanobacteria and chloroplasts (Kana, 1993; Asada, 2006). For instance, during N_2_-fixation period Mehler reaction consumes ~75% of gross O_2_ production and therefore maintains the O_2_ concentration at a low level (Kana, 1993; Milligan et al., 2007). However, Mehler reaction consumes reductant significantly (Asada, 2006); thus, in the future it will be of great interest and of vital importance to maintain the activity locally around the oxygen-sensitive enzymes rather than in the entire cytoplastic environment.

### SYSTEMATIC APPROACHES

#### Functional genomics

Functional genomics, i.e., transcriptomics, proteomics, and metabolomics, would greatly promote the development of synthetic biology in cyanobacteria and algae. Albeit the genomes of some many species of cyanobacteria and algae have been sequenced^1,2^, a large portion of the sequenced genomes have not yet been annotated and the regulatory networks are still very poorly understood. The study of cyanobacterial and algal transcriptomes, proteomes, and metabolomes would allow for identification of new genes, pathways and regulatory networks which are essential to expand the size and diversity of the pool of genetic tools for synthetic biology. For example, recent transcriptomics studies on *Synechocystis* 6803 has enhanced the understanding of the transcriptional regulation in this photosynthetic microorganism which revealed that approximate two-thirds of the transcriptional start sites give rise to asRNAs and noncoding RNAs (ncRNAs), indicating that asRNAs and ncRNAs play an important role in cyanobacterial genetic regulation (Mitschke et al., 2011). We prospect that omics may be the key to collect information about the interactions and regulations to develop a sustainable green chemistry industry.

#### Metabolic modeling

Although most synthetic biology research in cyanobacteria and algae focus on local pathway optimization, comprehensive synthetic biology summons optimization of the genetic network and metabolic flux at the systems level. Genome-scale metabolic modeling allows theoretically evaluating the impact of genetic and environmental perturbations on the biomass yield and metabolic flux distribution and allows predicting the optimal metabolic flux profile to maximize the value of a given objective function (Shastri and Morgan, 2005; Knoop et al., 2010; Yoshikawa et al., 2011). The *in silico* modeling may thus provide a systematic approach to design an optimal metabolic network to maximize the production of the interest biofuel or chemical. Such genome-scale metabolic network models have been constructed for cyanobacteria and algae, and have been utilized to predict new targets to improve product yields and new pathways (Shastri and Morgan, 2005; Knoop et al., 2010; Dal’Molin et al., 2011; Yoshikawa et al., 2011). However, the reconstruction of the global metabolic networks is still in the infancy stage and the simulation results rely significantly on the included pathways. For instance, with ambiguities in metabolic networks in *Synechocystis* 6803, the estimated metabolic fluxes could be significantly different from the experimental results (Yoshikawa et al., 2011). In order to refine the quality of the reconstructed metabolic networks and thus the simulation of metabolic flux, it is inevitable to couple with experimental characterization of the metabolic networks in cyanobacteria and algae (Yoshikawa et al., 2011). As an example, by firstly investigating the *in vitro* activities of the purified relevant enzyme products (heterologously expressed in *E. coli*) and subsequently verifying their *in vivo* activities in the native host *Synechococcus* 7002, Zhang and Bryant (2011) reported that two enzymes could functionally compensate for the missing 2-oxoglutarate dehydrogenase in the TCA cycle. Further database searches indicated that homologs of these two enzymes occur in all cyanobacteria but *Prochlorococcus* and marine *Synechococcus*, which overturned the previously widely accepted assumption that cyanobacteria possess an incomplete TCA cycle (Meeks, 2011; Zhang and Bryant, 2011). Such discoveries would be of utter importance to reconstruct qualified *in silico* models for simulating the metabolic flux in the future.

## CONCLUSION

Owing to the relatively simple genetic contents and the ability to capture solar energy, fix CO_2_, grow fast and directly synthesize specific products, cyanobacteria and algae have become excellent candidates for building autotrophic cell factories to produce renewable surrogate fuels and chemicals. With a large pool of genome sequences and improved genetic tools being available, application of synthetic biology in these photosynthetic microorganisms are highly desirable. In recent years, exciting results have been achieved not only in understanding of the fundamental molecular mechanisms but also in producing various interest products, such as biofuels and chemicals, utilizing cyanobacteria and algae as the production platforms. Nevertheless, synthetic biology in cyanobacteria and algae is still in its infancy and synthetic biologists are facing great challenges and opportunities in addressing various issues, such as improving the tools for genetic manipulation, enhancing light harvesting, increasing CO_2_ fixation efficiency, and overcoming of the intracellular oxidative stress. Systematic approaches, such as functional genomics and metabolic modeling, may also diversify the genetic tools and help the metabolic network design. It is doubtless that synthetic biology would be indispensable for the future success in applying cyanobacteria and algae for various biotechnological purposes.

## Conflict of Interest Statement

The authors declare that the research was conducted in the absence of any commercial or financial relationships that could be construed as a potential conflict of interest.

## Footnotes

1 http://www.genomesonline.org/

2 http://genome.jgi.doe.gov/

3 http://biobricks.org/

4 http://partsregistry.org/Part:BBa_B0015
